# Habitat quality is more important than matrix quality for bird communities in protected areas

**DOI:** 10.1002/ece3.3923

**Published:** 2018-03-25

**Authors:** Matti Häkkilä, Nerea Abrego, Otso Ovaskainen, Mikko Mönkkönen

**Affiliations:** ^1^ Department of Biological and Environmental Sciences University of Jyväskylä Jyväskylä Finland; ^2^ Department of Agricultural Sciences University of Helsinki Helsinki Finland; ^3^ Department of Biology Centre for Biodiversity Dynamics Norwegian University of Science and Technology Trondheim Norway; ^4^ Department of Biosciences University of Helsinki Helsinki Finland

**Keywords:** beta‐diversity, biotic homogenization, bird community, boreal forest, community composition, protected areas

## Abstract

Protected areas are meant to preserve native local communities within their boundaries, but they are not independent from their surroundings. Impoverished habitat quality in the matrix might influence the species composition within the protected areas through biotic homogenization. The aim of this study was to determine the impacts of matrix quality on species richness and trait composition of bird communities from the Finnish reserve area network and whether the communities are being subject of biotic homogenization due to the lowered quality of the landscape matrix. We used joint species distribution modeling to study how characteristics of the Finnish forest reserves and the quality of their surrounding matrix alter species and trait compositions of forest birds. The proportion of old forest within the reserves was the main factor in explaining the bird community composition, and the bird communities within the reserves did not strongly depend on the quality of the matrix. Yet, in line with the homogenization theory, the beta‐diversity within reserves embedded in low‐quality matrix was lower than that in high‐quality matrix, and the average abundance of regionally abundant species was higher. Influence of habitat quality on bird community composition was largely explained by the species' functional traits. Most importantly, the community specialization index was low, and average body size was high in areas with low proportion of old forest. We conclude that for conserving local bird communities in northern Finnish protected forests, it is currently more important to improve or maintain habitat quality within the reserves than in the surrounding matrix. Nevertheless, we found signals of bird community homogenization, and thus, activities that decrease the quality of the matrix are a threat for bird communities.

## 1 INTRODUCTION

Both theoretical (Lovejoy, 2006; Moilanen & Hanski, 1998) and empirical studies (Carroll, Noss, Paquet, & Schumaker, 2004; Newmark, 1996; Ricketts, 2001) support that protected areas are not independent from their surrounding matrix. The quality and quantity of the matrix surrounding isolated areas influence the rate of species loss (Prugh, Hodges, Sinclair, & Brashares, 2008; Sisk, Haddad, & Ehrlich, 1997), but we still know little about matrix effects on protected forest areas. Large areas can better maintain their species diversity because on small areas, the edge effect to area ratio is larger and the impact of the edge falls upon larger proportion of the area (e.g., Ries, Fletcher, Battlin, & Sisk, 2004). Rayner, Lindenmayer, Wood, Gibbons, and Manning (2014) demonstrated that species diversity within protected areas is highly sensitive to the quality of the matrix in which they are embedded. Correspondingly, Häkkilä et al. (2017) showed that in boreal bird communities, intensification of forest management in the matrix is associated with lowered species specialization, but increased functional diversity within the forest reserves.

The knowledge about the effects of matrix quality on the community differentiation (i.e., beta‐diversity) within protected areas is even more limited, albeit this knowledge is critical in conservation planning. Changes in beta‐diversity are not always reflected by changes in alpha diversity (Smart et al., 2006; Socolar, Gilroy, Kunin, & Edwards, 2016). Indeed, the quality of the matrix may affect the beta‐diversity within protected areas even if the alpha diversity remains unchanged. In such a case, conservation planning should take into account the structure of the landscape surrounding the protected areas.

Biotic homogenization refers to increasing similarity of biotic communities over space and time, and it is caused by nonrandom species extinctions and invasions due to human activities. Human land‐use intensification and changes such as urbanization (McKinney, 2006) and intensive agriculture (Ekroos, Heliölä, & Kuussaari, 2010) contribute to the homogenization process by diminishing rare and specialist species and promoting abundant and generalist species which are better able to cope in human‐altered environments (Clavel, Julliard, & Devictor, 2011; McKinney & Lockwood, 1999). This process is usually asymmetrical: few abundant and generalist species replacing a larger number of rare and specialist species (Devictor, Julliard, & Jiguet, 2008; McKinney & Lockwood, 1999; Morris & Heidinga, 1997). Consequently, biotic homogenization decreases both taxonomical and functional diversity over space and time (Clavel et al., 2011). Forest ecosystems are highly altered due to human activities (Secretariat of the Convention on Biological Diversity, 2010), yet little is known about whether and how forest‐dwelling communities suffer from biotic homogenization (but see Rooney, Wiegmann, Rogers, & Waller, 2004; Solar et al., 2015). In particular, boreal forests have been poorly studied, even if they represent 26% of the world's total forest area (Bryant et al., 1997) and are highly impacted by timber harvesting actions (Lundmark, Josefsson, & Östlund, 2013; Pohjanmies et al., 2017; Vanha‐Majamaa et al., 2007).

Biotic homogenization is a process that encompasses the loss of not only taxonomic diversity, but also its functional component (Olden et al. 2004). Due to biotic homogenization, communities become functionally more similar, ultimately affecting ecosystem functioning (Hooper et al., 2005). Furthermore, analyzing community composition in terms of functional traits can be more informative than focusing on species identities, as they can inform about the ability of the species to adapt to particular environmental characteristics (Cadotte, Carscadden, & Mirotchnick, 2011). In the case of birds, it has been shown that resident species are more vulnerable to anthropogenic changes than migratory species (Imbeau, Mönkkönen, & Desrochers, 2001), because resident birds are dependent on habitat resources all year round, whereas migratory birds only visit when the resources are most abundant (Mönkkönen & Welsh, 1994). Morphological traits of birds are well known to be associated with their diet, and movement and foraging behavior (Carrascal, Moreno, & Telleria, 1990; Jønsson, Lessard, & Ricklefs, 2015; Miles & Ricklefs, 1984). For instance, body size is associated with extinction risk, because larger species tend to have lower fecundity, and thereby higher sensitivity to habitat disturbances (Bennett & Owens, 1997). Using traits in our analyses, it is possible to study which characteristics are particularly sensitive to environmental change and thereby to reveal the mechanisms of biotic homogenization.

In intensively managed Fennoscandian boreal forests, protected areas are surrounded by young, fast‐growing forests. Some forest‐dependent bird species benefit from logging in the matrix by foraging in the matrix (Jokimäki & Huhta, 1996), whereas others are strictly confined to old‐growth forests. The managed forest matrix may thus alter the community composition within protected areas by benefitting the occurrences of more generalist species that make use of the resources in the matrix. Correspondingly, Mönkkönen, Rajasärkkä, and Lampila (2014) found that the number of specialist bird species is lower in old‐growth forest patches surrounded by managed forests than in continuous old‐growth forests. The effects of the matrix quality may additionally differ in relation to the size of the protected areas. The effects of the matrix quality may be particularly acute in small protected areas (Carroll et al., 2004) because small area renders it difficult to maintain viable populations (Gaston, Jackson, Cantú‐Salazar, & Cruz‐Piñón, 2008).

The aim of this study was to determine whether the matrix quality impacts the species richness and trait composition bird communities and whether the Finnish protected area network suffers from biotic homogenization due to matrix effects. To address these aims, we use an extensive dataset of bird occurrence data comprising 69 species in 91 nature reserves in northern Finland. We apply a hierarchical joint species distribution model to simulate bird community scenarios in forest reserves of different sizes, habitat quality, and matrix quality. Specifically, we ask whether the matrix quality affects (1) the taxonomical community composition, (2) functional trait composition, and (3) community similarities (beta‐diversity) within forest reserves when these differ in size, habitat, and matrix quality.

If homogenization occurs, reserves embedded in matrices with high level of disturbance (high proportion of shrubs and saplings) will have lower beta‐diversity than those embedded in less disturbed matrix. Homogenization effects will be strongest in reserves where differences in habitat composition between reserves and the surrounding matrix are the greatest. We further hypothesize that the matrix effects will be strongest on small reserves, whereas large reserves are better able to maintain their integrity. If so, conservation efforts should be focused on large areas, and in case of small areas, on managing the surrounding matrix to minimize contrast to the protected areas in landscape structure. We also expect changes in species traits with changes in habitat quality in the reserves and in their surrounding matrix. In particular, we hypothesize that resident species, specialist species as well as species with large body size will be especially susceptible to disturbance in matrix.

## 2 METHODS

### 2.1 Study area

**Figure 1:**
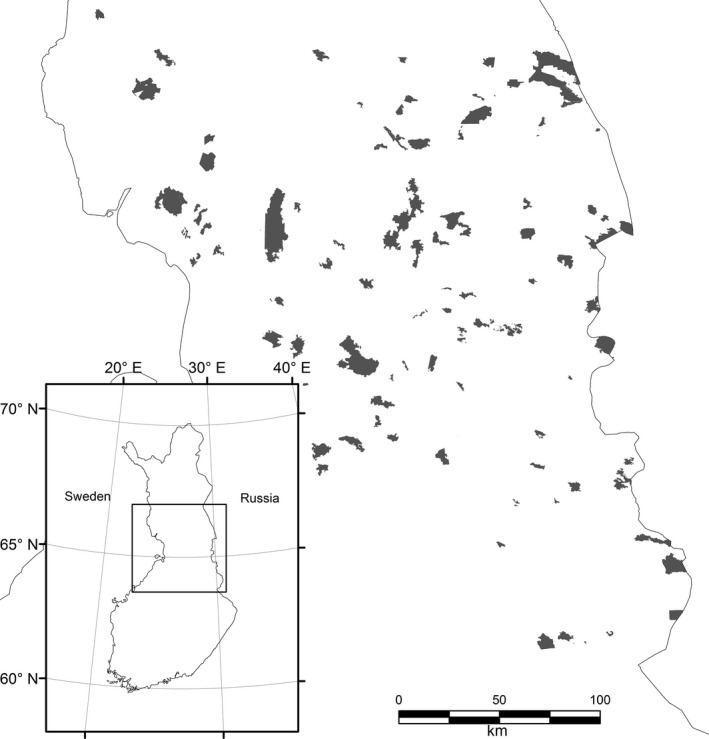
Map of the study area and the location of the forest reserves in northern Finland

The study area is in the boreal zone in northern Finland (Figure 1) where forests are mainly coniferous (Table 1). The area is sparsely populated and dominated by forest land, but open bogs, mires, small lakes, and ponds are characteristics of the landscape. Most forests are intensively managed. This study focuses on 91 unmanaged nature reserves with a total area of approximately 3,100 km^2^. Reserves vary in size from 200 to 28,000 ha (mean area = 3,400 ha, *SD* = 4,676 ha), and their average distance to nearest neighbor area was 13,047 m.

**Table 1:** Percentages of the cover types inside the protected areas and their matrices. The average, minimum and maximum percentages are shown

Cover type	Abbreviation of habitat type	Inside			Matrix		
		$\overset{¯}{x}$	Min	Max	$\overset{¯}{x}$	Min	Max
Pine‐spruce >100 m^3^/ha %	1	16.9	0.8	56.2	7.2	0.9	25.1
Pine >100 m^3^/ha %	2	3.3	0.1	19.0	2.6	0.3	8.6
Spruce‐Deciduous >100 m^3^/ha %	3	6.8	0.2	38.6	3.2	0.5	10.0
Spruce 25—100 m^3^/ha %	4	12.4	1.4	34.6	9.9	2.5	21.2
Pine 25—100 m^3^/ha %	5	10.9	0.4	27.3	13.1	3.9	25.0
Spruce‐Deciduous 25—100 m^3^/ha %	6	10.7	1.9	45.0	14.4	5.4	33.9
Pine bogs %	7	20.0	0.3	50.9	18.6	7.3	33.2
Shrubs <25 m^3^/ha %	8	5.7	0.7	17.9	15.1	5.4	32.9
Other open areas %	9	13.3	0	35.9	15.8	7.1	26.4

### 2.2 Data description

#### 2.2.1 Environmental data

Land use and cover data were acquired from the 8th National Forest Inventory of Finland (NFI) for which the data were collected 1986–1994 (Tomppo, 1993). These multisource data are based on satellite images and their interpretation, and entirely cover the protected areas and their matrices. Data resolution is 25 m × 25 m. For each pixel of forested land, NFI produces an estimate of growing stock volume separately for *Pinus sylvestris*,* Picea abies*,* Betula spp*., and all other species as a combined class. Digital maps of nonforest (peatland, water, agricultural land, roads, and settlements) lands are used to separate nonforest areas from forest. Using these data, land cover of the study area was classified into nine classes according to vegetation structure (see Brotons, Mönkkönen, Huhta, Nikula, & Rajasärkkä, 2003; Table 1). From this classification, we calculated variables describing the habitat composition within the reserves and in the surrounding landscape. We used the sum of spruce‐deciduous cover types (habitat classes 3 and 6; Table 1) to describe productivity. In the study area, spruce‐deciduous forests only grow on fertile soil while less fertile sites are usually pine‐dominated. To describe the proportion of old forest, we used the sum of the three cover types with more than 100 m^3^/ha (habitat classes 1–3; Table 1). On advanced thinning stands, where most trees have reached saw timber size, the average stock volume in northern Finland is 118 m^3^/ha, but in southern Lapland only 99 m^3^/ha (Peltola, 2014). Therefore, we chose 100 m^3^ as a limit above which we consider forest old. We assume that the proportion of forests with saw timber stock within reserves is related to their habitat quality because we focus on forest birds. We analyzed matrix quality within 5 km radius around the reserves. 5 km radius was selected to make sure that the matrix could have impact on species with large home ranges such as large raptors. Larger radii could have resulted in an excessive overlap in matrices of neighboring areas. A portion of the matrices around reserves adjacent to or near the Russian border fell outside the Finnish land‐cover data, and comparable data from Russia were not available. In such cases, landscape structure in the buffer zone was estimated assuming that undisturbed areas along the Russian border have identical landscape composition compared with the reserve itself. This assumption is reasonable because Finnish forest reserves represent natural, undisturbed areas corresponding to the state of forests along the Russian side of the border. As indicator for low quality, we used the proportion of shrubs and saplings (habitat class 8; Table 1), because intense clear‐cutting activities result in landscapes dominated by young trees.

#### 2.2.2 Bird data

The bird species abundance was measured with the Finnish line transect census method (Järvinen & Väisänen, 1976) by Metsähallitus Parks & Wildlife Finland. Because the basic idea was to study whether species living in protected areas are safeguarded from the impacts of logging we focused on forest species, and thus of the 129 species in the original data, including wetland species, we selected 69 species known to use forest as their main breeding habitat (Väisänen, Lammi, & Koskimies, 1998). The bird censuses were conducted between 1988 and 1999 for a total of 3,323 km of transects. On average, 1 km of transect per km^2^ of land area was surveyed; small areas being surveyed with higher per‐unit‐area effort. We combined data across years as earlier analyses showed that species richness and abundance of forest species in these data did not differ significantly among years (Brotons et al., 2003).

#### 2.2.3 Trait data

We compiled data on morphological traits, migratory patterns, habitat requirements, and population characteristics (Table 2). We made morphological measurements (wing, tail, tarsus and bill length, bill width, bill height, and body mass) of museum samples of a minimum of five individuals per species. As all of these morphological measures are strongly correlated and reflect the body size of the bird, we transformed original morphological variables into indices that link morphology with ecological functions. First, we used body mass (log‐transformed) as an indicator of overall body size. Body size is important driver of both habitat use and diet. Second, to describe functions related to the type of food, we used a ratio bill length/(bill width + bill height). Species with long bills relative to bill width and height tend to be more insectivorous than short‐billed species (Lederer, 1975). Third, we calculated three further ratios (wing length/body mass ^1/3^, tarsus length/body mass ^1/3^, and tail length/body mass ^1/3^) to represent differences in locomotion and habitat use (Miles & Ricklefs, 1984). The lengths were divided by the cubic root of body mass to scale these one‐dimensional variables by a one‐dimensional measure of body size.

**Table 2:** Description of the traits included in the analyses

Trait	Description	Units
Morphological traits		
Log‐transformed body size	Body mass (g)	Continuous
Bill ratio	Bill length/(bill width + bill height)	Continuous
Wing length	Wing length/(body mass^1/3^)	Continuous
Tarsus length	Tarsus length/(body mass^1/3^)	Continuous
Tail length	Tail length/(body mass^1/3^)	Continuous
Migratory patterns		
Resident, migratory	Whether the species are resident or migratory (either long‐ or short‐distance)	Categorical, two levels
Habitat requirements		
SSI	Species (habitat) specialization index	Continuous
Population characteristics		
Population size	Minimum count of breeding bird pairs in Finland	Continuous
Population trend	Whether the bird populations have increased, decreased, or remained stable during the last 20–30 years in Finland.	Categorical, three levels

We classified the bird species as resident or migratory (either long‐ or short‐distance) according to Svensson, Grant, Mullarney, and Zetterström (2010). We used the species specialization index (SSI) as a measure of habitat specialization (Julliard, Clavel, Devictor, Jiguet, & Couvet, 2006). For the calculation of SSI, we used Finnish point count data that were collected 1984–2011 (Laaksonen & Lehikoinen, 2013). The observations in the data are categorized into 17 habitat classes Koskimies & Väisänen, 1991) from which we calculated the coefficient of variation (standard deviation/average among habitat classes) for each species. We used the estimated minimum count of breeding pairs in Finland as a measure of the population size (Valkama, Vepsäläinen, & Lehikoinen, 2011). We also considered the population trends of the species in Finland. For the latter one, we used the Finnish bird atlas (Valkama et al., 2011) and classified the species as increasing, decreasing, or stable population trends.

### 2.3 Statistical modeling

#### 2.3.1 Model fitting and assessment of model fit

The original data consist of counts of 69 bird species on transects ranging from 1 to 234 km per reserve. For getting comparable sampling units, we divided the transects into 1 km segments, the smallest length of the original transects, randomly assorted the counts of each species to the segments, and then transformed the data to presence–absence within segments (see Supporting Information for more details). The transformed dataset consisted of presence–absence data of the 69 bird species in 2,500 segments nested within the 91 reserves.

We analyzed the presence–absence of the bird species at the level of 1 km segments by fitting a joint species distribution model with the HMSC Matlab‐package (Ovaskainen et al., 2017). We used probit regression to model species occurrence probabilities at each 1 km segment. As explanatory variables, we included (1) the log‐transformed area of the reserves, (2) the indicator of the habitat productivity within reserves (proportion of productive forest types), (3) the proportion of old forests within reserves, and (4) the proportion of shrubs (clear‐cuts) in the matrix. To examine the joint influence of matrix and habitat quality within reserves, we also included (5) the interactions between variables 1 and 4, and (6) the interaction between variables 3 and 4. To account for the nested structure of the data (i.e., segments nested within reserves), we included the reserve id as a random effect. We incorporated into the model species traits to examine how much of the variation in species occurrences was be explained by traits. As traits we included those described in Table 2.

We assessed how accurately the model predicted species occurrences at the level of segments by performing cross‐validation, where we refitted the model 91 times so that each time we excluded the data from one of the 91 reserves. We used these models to predict the posterior mean occurrence probability of each species for the reserve that was excluded for model fitting. We then computed for each species the correlation (over the reserves) between the predicted occurrence probabilities and the fraction of segments in which the species was observed. We averaged the species‐specific correlations over the species to obtain an overall measure of the model's predictive power. We followed the procedure of Abrego, Norberg, and Ovaskainen (2017) to partition the explained variation among the environmental covariates and random effects, and to assess how much of the variation in species occurrences is be explained by their traits.

#### 2.3.2 Assessing the influence of reserve size, habitat quality within reserves, and matrix quality on bird community composition

We used scenario simulations to examine how reserve area, reserve quality, and matrix quality influence species density (number of species/1 km transect) and community composition. For this, we created eight scenarios for which we varied systematically the size of the reserve and its habitat quality, as well as buffer quality (Table 3), and by simulation predicted the occurrence probabilities of the species for 1 km transect segments.

**Table 3:** Scenarios used to examine how bird community structure and trait distribution are influenced by the size of the reserves, its habitat quality and the quality of the buffer area. In the symbols, green color denotes high‐quality habitat and red low‐quality habitat

Scenario	Symbol	Environmental conditions
Baseline: large area, high quality inside and outside	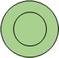	Large reserve with high proportion of old forests and low shrub proportion in the matrix.
Small, low quality inside and high quality outside	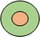	Small reserve with low proportion of old forests and low shrub proportion in the matrix.
Small, low quality inside and low quality outside	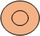	Small reserve with low proportion of old forests and high shrub proportion in the matrix.
Small, high quality inside and high quality outside	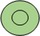	Small reserve with high proportion of old forests and low shrub proportion in the matrix.
Small, high quality inside and low quality outside	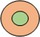	Small reserve with high proportion of old forests and high shrub proportion in the matrix.
Large, low quality inside and high quality outside	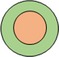	Large reserve with low proportion of old forests and low shrub proportion in the matrix.
Large, low quality inside and low quality outside	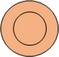	Large reserve with low proportion of old forests and high shrub proportion in the matrix.
Large, high quality inside and low quality outside	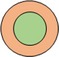	Large reserve, with high proportion of old forests and high shrub proportion in the matrix.

We considered the predicted community composition within large reserve with high‐quality habitat, surrounded by a high‐quality matrix as the reference baseline scenario. We defined “small” and “large” reserve areas as the 10% and 90% quantiles of the distribution of reserve areas in the data, “low” and “high” proportions of old forest as the 10% and 90% quantiles of the distribution of proportion of old forest, and “low” and “high” proportions of shrubs as the 10% and 90% quantiles of distribution of proportion of shrubs. High‐quality matrix corresponds to low proportion of shrubs, and vice versa. Productivity was set to its mean value for all simulated forests.

For each of the scenarios, we generated 100 simulated communities, for each of which we sampled the model parameters from the posterior distribution. For each of the eight scenarios, we predicted the expected species density, as well as community similarity to the reference community. We note that one of the scenarios (large high‐quality reserve with high‐quality buffer) is identical to that of the reference scenario (Table 3). Thus, community similarity between these two scenarios describes the amount of natural variation in community structure.

We assessed the influence of (1) reserve quality, (2) reserve area, and (3) matrix quality on the community composition by computing the posterior probabilities that (1) the community in a high‐quality reserve is more similar to the natural reference community than a community in low‐quality reserve separately for the four cases corresponding to a small versus large reserve, and low‐ versus high‐quality matrix, (2) the community in a large reserve is more similar to the natural reference community than a community in a small reserve, separately for the four cases corresponding to a low‐ versus high‐quality reserve, and low versus high‐quality matrix, and (3) the community in a reserve surrounded by high‐quality matrix is more similar to the natural reference community than a community surrounded by low‐quality matrix, separately for the four cases corresponding to a small versus large reserve, and low‐ versus high‐quality reserve.

#### 2.3.3 Assessing the influence of reserve size, habitat quality within reserves, and matrix quality on functional bird community composition

To characterize the functional composition of bird communities in each simulated scenario, we converted the predicted data on species compositions to trait compositions. We did this by averaging the values of each trait category over the species predicted in each scenario. We computed the mean trait values for 100 replicate communities in each of the eight scenarios. We then computed the posterior mean of mean trait values in each scenario and the posterior probability that the mean trait value was lower in a particular scenario than in the reference scenario.

#### 2.3.4 Testing the homogenization hypothesis

To address the homogenization hypothesis, that is, that similarity in community composition among reserves increases with increasing human impact in the matrix, we defined the following homogenization measure. We let *p*_*ij*_ denotes the occurrence probability of species *j* under a scenario *i*. Then $V_{\mathrm{ij}}=p_{\mathrm{ij}}\left(1-p_{\mathrm{ij}}\right)$ corresponds to the variance of the Bernoulli distributed random variable which models the occurrence of the species. We define *V*_*i*_ as the mean value of the *V*_*ij*_ over all species, and call it the community variability under the scenario *i*. If *V*_*i*_ = 0, then the community compositions are deterministic: Some species are present with certainty and others absent with certainty, meaning that there is a maximal level of within‐scenario homogenization. If *V*_*i*_ = 0.25, then the community compositions are as variable as possible: Each species is present with probability 0.5, meaning that there is little homogenization. We computed the community variability *V*_*i*_ for all scenarios, and computed the posterior probability by which community variability was lower than for the reference scenario. In their relatively similar approach, Baeten et al. (2014) use the sum of *V*_*ij*_ instead of mean, but these two approaches give identical inference and thus the posterior probability by which community variability was lower for each scenario than for the reference scenario is identical whether it is computed for sum or mean.

When interpreting the outcomes of the models, we considered >0.95 posterior probabilities providing strong statistical support and posterior probabilities 0.90–0.95 providing support, but not strong, to our hypotheses.

## 3 RESULTS

### 3.1 Overall bird community composition

Based on the cross‐validation, the fitted model explained 50% of the variation in bird species occurrence probabilities (averaged over the species) at the level of 1 km segments. Out of this variation, the environmental covariates explained 70%, and the random effects (i.e., reserve id) 30% (Figure S1). Most of the explained variation (56%) was attributed to the size of the reserve, the reserve quality, the matrix quality, and the interaction between the latter two. The productivity of the forests explained the remaining 14% of the variation.

### 3.2 Influence of reserve area, reserve quality, and matrix quality on bird community composition

The bird communities most similar to the communities in the reference scenario were those from the scenarios which had a high‐quality habitat within the reserve, regardless of the matrix quality and the size of the reserve (Table S1). This suggests that the habitat quality within reserves is the main driving force to community composition. Accordingly, we found that the similarity between the reference community and a community in a high‐quality reserve was greater than the similarity between the reference community and a community in a low‐quality reserve, irrespective of the size of the reserve and matrix quality (Table 4, 1st row, Table S2). Reserve area (Table 4, 2nd row; S2) and matrix quality (Table 4, 3rd row; Table Table S2) did not have a substantial influence on the community composition, as the posterior probabilities for all comparisons related to these variables varied from 0.1 to 0.88.

**Table 4:** Effects of reserve quality, reserve area, and matrix quality on bird community composition. The effects have been measured by computing the posterior probabilities that the communities in “A” scenarios are more similar to the baseline reference scenario (i.e., large high‐quality reserve surrounded by a high‐quality matrix) than “B” scenarios. The cases in which the posterior probability is >0.95 are indicated by darker yellow, cases in which the posterior probability is ≥to 0.90 by lighter yellow and the cases in which the posterior probability is <0.90 are in white. The numerical values of the similarity measure for each of the scenarios are provided in Table S1, and the numerical values of the posterior probabilities used to construct the figure are provided in Table S2. The symbols are the same as in Table 3

Effect of patch quality	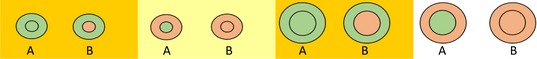
Effect of patch size	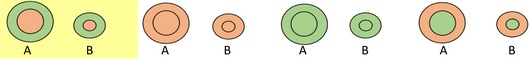
Effect of matrix quality	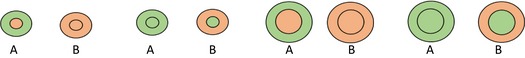

We did not have strong support for matrix effects being stronger in small reserves. For example, similarity of a community in a small reserve with high‐quality habitat but surrounded by low‐quality matrix with the reference scenario was equal to that of a large reserve with otherwise similar characteristics (similarities 0.86 vs. 0.89, Table S1; posterior probability for difference ≪0.9); were the matrix effects stronger in small reserves, we would have observed lower similarity for a small than a large reserve.

The expected species density was highest (15.8 species/1 km segment) in small high‐quality reserves surrounded by high‐quality matrix (Table 5). The expected species density was very similar (13.2–14.4) among all the remaining seven scenarios. We did not find any statistical support for differences in species density between the reference and other scenarios (posterior probability for differences ≪0.9; Table S3).

**Table 5:**
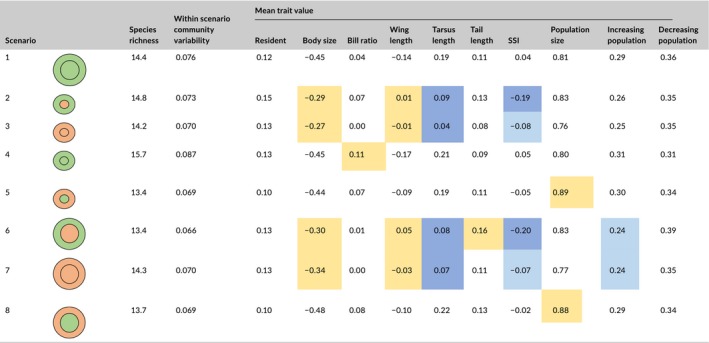
Predicted species density, within‐scenario variability in community composition (beta‐diversity), and bird trait composition in each of the simulated scenarios. The numerical values correspond to the posterior means for the expected species density, community variability, and mean trait values in each of the scenarios. The colors of the cells indicate whether the trait value is larger (yellow color) or smaller (blue color) than in the reference scenario with >0.95 (darker color) and with ≥0.90 posterior probability (lighter color). The numerical values of the posterior probabilities are provided in Table S3. The symbols are the same as in Table 3

### 3.3 Functional bird community composition

The traits included in the model explained 52% of the variation explained by the environmental covariates. Compared to the reference scenario, the body size of the birds was larger in those scenarios which have low reserve quality (≥ 0.90 posterior probability for the four scenarios with low reserve quality, Table 5). Likewise, the relative wing length was larger and the relative tarsus length smaller in those scenarios which have low reserve quality (≥ 0.90 posterior probability in all cases, Table 5).

The average specialization index for the habitat was smaller in those scenarios which have low reserve quality (≥ 0.90 posterior probability for the four scenarios with low reserve quality), but we found no support for the hypothesis that low‐matrix quality is associated with low level of specialization (Table 5). The average population size of the species in the communities was highest in those scenarios of high‐quality reserves surrounded by a low‐quality matrix (posterior probability ≥ 0.90, Table 5) suggesting that low‐quality matrix is associated with an increased abundance of common species in high‐quality reserves. Those bird species with an increasing population trend were less prevalent in scenarios with large‐ but low‐quality reserve (posterior probability ≥ 0.90, Table 5).

### 3.4 Community homogenization

The results provide support for the hypothesis that matrix quality is associated with homogenized bird community composition within reserves. Posterior mean of community variability was highest for the scenario with a small high‐quality reserve surrounded by a high‐quality matrix and second highest for the reference scenario (scenarios 1 and 4 in Table 5). Lowest beta‐diversity values were expected for the high‐quality (small and large) reserves embedded in low‐quality matrix (scenario 5 and 8 in Table 5). Even though both values (0.069) are among the smallest ones, their difference from the reference scenario did not gain strong statistical support (Table 5). The lowest value was observed for the scenario where large low‐quality reserve is surrounded by a high‐quality matrix, and this differed from the reference scenario with posterior probability 0.94.

## 4 DISCUSSION

We observed a relatively small effect of the matrix quality on the composition of bird communities, whereas the quality of the habitat within the forest reserves strongly influenced the bird community composition. Interestingly, the variation in bird community composition in the reserves was largely explained by the species' functional traits. The community specialization index was low, and average body size was large in reserves with low proportion of old forests. Even though the matrix quality did not strongly influence the bird community composition within the reserves, we found some signals of community homogenization associated with low‐quality matrix. The beta‐diversity within reserves was lower (yet with low statistical support) if the reserves were embedded in low‐quality matrix than in high‐quality matrix. Importantly, in line with the homogenization theory, we found that in reserves situated in low‐quality matrix, regionally more abundant species became more abundant.

### 4.1 Community composition

As expected, the proportion of old forest within the reserves was the main factor in explaining the bird community composition. However, in contrast to results from earlier studies (Devictor, Julliard, Clavel, et al., 2008; Kennedy, Marra, Fagan, & Neel, 2010; Stouffer, Strong, & Naka, 2009), our results showed only moderate responses of bird communities to the quality of the matrix. Furthermore, the responses of bird communities were not stronger in small reserves. These results might be attributed to the design and scale of the study. First, our study units (i.e., reserves) were on average larger than in the precedent studies (our study units were 34 km^2^ on average, whereas Devictor, Julliard, and Jiguet (2008) used 4 km^2^ study units and Kennedy et al. (2010) worked on 1 km^2^ study units). Thus, our results suggest that larger high‐quality areas might be better buffered against the matrix effects (Carroll et al., 2004). Other plausible explanation is that the quality of the matrix in Finnish northern forest reserves is not contrasting enough. In studies in which strong matrix effects were found (Kennedy et al., 2010; McKinney, 2006), the difference in the habitat quality between the focal areas and the matrix was greater (native vs. urban habitats) than in our study. In our case, the lowest matrix quality belonged to recently logged forests, which basically represent forests in the very early successional stage. In our case, the matrix is not totally inhabitable, and even some old forest specialists are able to use resources in the matrix (Mönkkönen et al., 2014).

### 4.2 Species density

We found no statistically supported differences in species density in larger areas compared with small. Thus, larger reserves do not contain more species per unit area even if total species richness increases with the area of reserves (Häkkilä et al., 2017). In contrast, we found the highest predicted species density for the scenario of small area with high proportion of old forests and low proportion of shrubs around. This may stem from the spillover effect from the surrounding matrix (landscape supplementation, sensu Dunning, Danielson, & Pullian, 1992). Even the highest‐quality matrix contained more early and mid‐successional forests than most of the reserves (Table 1), and therefore fostered more species associated with early‐ and mid‐successional forests. Our result does not support earlier findings from the same region by Mönkkönen et al. (2014) who found no area effects on total bird species richness per standard sample size (# individuals). Mönkkönen et al. (2014) found a clear matrix effect so that for a given sample size, remnant old forest patches in human‐modified landscapes foster fewer species than old forests embedded in intact forest landscapes. In summary, it seems that in these boreal settings, highest species densities are found in pristine landscapes (see also Edenius & Elmberg, 1996) but in human‐dominated landscapes, small reserves may have the highest species densities due to the spillover.

### 4.3 Functional composition

Our results showed that the variation in bird community composition in the forest reserves varying on habitat and matrix quality was largely explained by the species' functional traits.

First, we observed a clear pattern on the variation of morphological traits. Areas with small proportion of old forest hosted species with larger body size and longer relative wing length but shorter relative tarsus. This can be an outcome of higher abundances of raptor and grouse species (Rayner, 1988) in areas with more habitat variation and also opens areas such as bogs. We also observed larger bill ratio in small, forested areas with only little shrub habitats in the matrix. This reflects increasing abundance of small‐sized insectivore birds such as warblers and tits (Miles & Ricklefs, 1984) in small reserves with high old forest cover and high‐quality matrix.

Second, reserves with low old forest cover showed lower average species specialization index (SSI) values irrespective of the matrix quality. This indicates that habitat quality within the reserves affects the relative abundances of specialist and generalist species, reserves with high old forest cover harboring more specialist species than reserves with low old forest cover. This result supports previous studies showing that habitat disturbance favors generalists at the expense of habitat specialists (e.g., Clavel et al., 2011; Devictor, Julliard, & Jiguet, 2008; Marvier, Kareiva, & Neubert, 2004).

Third, the abundance of species with nationally large population size was higher in a high‐quality area surrounded by low‐quality matrix. Therefore, community homogenization due to low‐quality matrix occurs by the increase of common or abundant bird species.

### 4.4 Community similarity

The results provide some support for the hypothesis that matrix quality is associated with larger community similarity (homogenization) within reserves because we found the highest community variability (beta‐diversity) values in scenarios where high‐quality reserves (both small and large) were embedded in high‐quality matrix. Conversely, high‐quality reserves in low‐quality matrix showed beta‐diversity values that were among the lowest ones. Thus, communities in reserves embedded in low‐quality matrix are more similar to each other than those embedded in high‐quality matrix, as predicted by the biotic homogenization hypothesis. We also found that in high‐quality reserves surrounded by low‐quality matrix the species specialization did not differ statistically from the reference scenario and that average population size of species was higher. Homogenization likely originates from more common, abundant species becoming more pervasive in the reserves embedded in low‐quality matrix. This may also result from landscape supplementation effect (sensu Dunning et al., 1992), that is, a spillover into the reserves of abundant species from the surrounding matrix. We found little evidence for the prediction that small reserves will be particularly sensitive to a decrease in community variability. Thus, large size may not buffer reserves against negative matrix effects, and maintaining high matrix effects may be important irrespectively of the reserve size.

## 5 CONCLUSIONS

Our results show that for conserving local bird communities in northern Finnish forest reserves, it is more important to focus on improving or maintaining the habitat quality within the reserves than in the surrounding matrix. However, we note that this study concentrates only on birds that have relatively good dispersal ability, and the responses could be different in other species groups. Furthermore, we found signals of bird community homogenization due to impoverished matrix quality. Thus, if the quality of the matrix is not considered in conservation planning, this may compromise the ability of a conservation area network in maintaining local communities.

## CONFLICT OF INTEREST

None declared.

## AUTHORS' CONTRIBUTION

MH (corresponding author) involved original idea, conceptualization, writing, results interpretation; NA involved in conceptualization, analysis, visualization, results interpretation, revising; OO involved in conceptualization, analysis, result interpretation, revising; MM involved in original idea, conceptualization, revising, results interpretation. All authors contributed critically to the drafts and gave final approval for publication.
