# Supplementary material for: Habitat quality is more important than matrix quality for bird communities in protected areas
Source: Ecol Evol. 2018 Mar 25;8(8):4019–30. doi: 10.1002/ece3.3923 (PMC5916276; doi:10.1002/ece3.3923)
Supplement: Supplementary file 1 [file ECE3-8-4019-s001.docx]

**
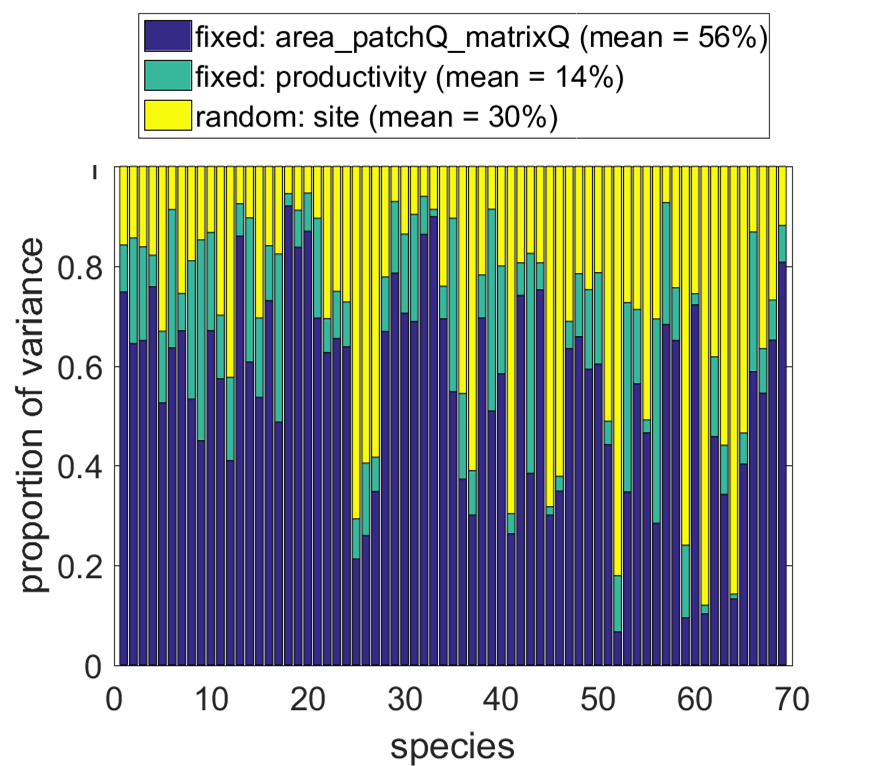
**

*Figure S1. The relative proportions of variance in species occurrence attributed to the fixed effects and to the random effects. The variance attributed to the fixed effects (measured covariates) is shown by bluish colors, whereas the random effects are shown by yellow.*
